# Pro12Ala Polymorphism on the PPAR_γ_2 Gene and Weight Loss After Aerobic Training: A Randomized Controlled Trial

**DOI:** 10.3389/fphys.2020.00385

**Published:** 2020-05-08

**Authors:** Glêbia Alexa Cardoso, Darlene Camati Persuhn, Mateus Duarte Ribeiro, Bruno Rafael Virgínio de Sousa, Klécia de Farias Sena, Antônio Eduardo Monteiro de Almeida, João Modesto-Filho, Raquel Suelen Brito da Silva, Alexandre Sérgio Silva

**Affiliations:** ^1^Laboratory of Applied Studies in Physical Training to Performance and Health - LETFADS, Department of Physical Education, Federal University of Paraíba, João Pessoa, Brazil; ^2^Associate Graduate Program in Physical Education – UPE/UFPB, Department of Physical Education, Federal University of Paraíba, João Pessoa, Brazil; ^3^Graduate Program in Nutrition Sciences, Federal University of Paraíba (PPGCN/UFPB), João Pessoa, Brazil; ^4^Lauro Wanderley University Hospital – HULW-Federal University of Paraíba – UFPB, João Pessoa, Brazil

**Keywords:** body composition, genetic polymorphism, PPARγ2, aerobic exercise, weight loss

## Abstract

The objective of this study was to verify the influence of the Pro12Ala polymorphism of the PPARγ2 gene in response of a training program on the body composition. Sixty-nine previously inactive men and women (32.8 ± 8.2 years) were genotyped and underwent a 12-week aerobic (running/walking) training program (3–5 sessions, 40 – 60 min per session, and intensity between the aerobic and anaerobic threshold) (experimental group *n* = 53) or were part of the control group (*n* = 16). They were tested for aerobic capacity (ergospirometry), body composition (DXA), abdomen, waist and hip circumferences and nutritional assessment before and 48 h after the experimental protocol. Two-way repeated measures ANOVA test was used to verify possible differences in variables between the experimental vs. control groups or Pro/Pro vs. Pro/Ala groups, and the Chi-squared test was used to verify the distribution of responders and non-responders according to genotype (*p* < 0.05). Frequencies of 75.5% Pro/Pro (*n* = 40) and 24.5% Pro/Ala (*n* = 13) were found, without any occurrence of the recessive homozygote. Body fat reduction was initially confirmed compared to a control group which did not exercise (*n* = 16; 29.1 ± 8.8 years), so that the exercise group obtained a reduction of −1.3 kg vs. −0.3 kg in the control group (*p* = 0.03). When they were divided by genotype, there were significant changes in fat mass (−1.3 ± 2.1 kg; *p* = 0.00), lean mass (0.6 ± 1.5 kg; *p* = 0.02), fat percentage (−1.3 ± 1.6; *p* = 0.00), waist circumference (−2.2 ± 2.9 cm; *p* = 0.00), abdomen circumference (−3.3 ± 3.6 cm; *p* = 0.00) and hip circumference (−2.7 ± 2.7 cm; *p* = 0.00) for Pro/Pro genotypes; and fat mass (−1.1 ± 1.7 kg; *p* = 0.04), fat percentage (−0.9 ± 1.5; *p* = 0.04), abdomen circumference (−3.9 ± 3.5 cm; *p* = 0.00) and hip circumference (−1.8 ± 1.8 cm; *p* = 0.00) for Pro/Ala genotypes, without any group interaction differences. The Chi squared test revealed no differences in the distribution of responders or non-responders according to genotype. It is concluded that an aerobic training program promotes weight loss, but the Pro12Ala polymorphism in the PPARγ2 gene does not influence the variability of aerobic-induced exercise weight loss.

## Introduction

Despite scientific advances, weight loss remains a challenge regardless of intervention strategy. A meta-analysis indicates that interventions with diets and lifestyle changes promote a reduction of around 5 kg after 2–4 years, while pharmacological therapies result in a reduction of 5–10 kg after 1–2 years (Douketis et al., 2005). Meta-analytic studies of the physical training practiced alone (without dietary or pharmacological intervention) indicates weight loss from 0.4 ± 3.3 kg to 2.3 ± 5.5 kg (Johns et al., 2014) or 0.9–2.9 kg (Washburn et al., 2014).

An important individual variability in physical training responses has been noted with people who are good or bad responders and who even acquire body fat after training programs (Donnelly et al., 2003). In a randomized controlled clinical trial of walking/running for 10 months (5 sessions/week, 400 or 600 kcal/session) (Donnelly et al., 2013), only 62.2% of those who spent 600 kcal/session and 45.9% to 400 kcal/session achieved ≥ 5% weight loss compared to baseline, while the other participants did not lose or even gained body weight. Therefore, understanding differences between good and bad responders to training programs can provide important insights for this line of research.

The gamma peroxisome proliferator-activated receptor gene (PPARγ2) may be considered a candidate gene for weight loss. Previous data indicate that this gene is involved in adipocyte regulation, growth and differentiation (Fajas et al., 1997), regulates the expression of numerous genes involved in lipid metabolism, controls the expression of the fatty acid carrier protein, and is predominantly expressed in adipose tissue (Tavares et al., 2007). In fact, this gene is related to obesity and metabolic diseases (Masud et al., 2003; Ereqat et al., 2009; Bozina et al., 2013; Galbete et al., 2013).

The influence of this polymorphism on weight loss is still controversial. Studies have shown that patients with the Ala12 allele of the PPAR gene had greater weight loss in response to a training program (150 min of physical activity/week) in the short and long term (0.63 and 0.93 kg/allele, *p* < 0.005, respectively) (Delahanty et al., 2012). Moreover, diabetic patients with the Pro/Ala + Ala/Ala allele presented a higher body weight reduction when compared to Pro/Pro homozygotes, who underwent exercise intervention (−1.8 ± 1.8 kg vs. −0.3 ± 1.4 kg) (Østergård et al., 2005). On the other hand, women with Pro12Ala polymorphism showed resistance to a dietary intervention (Adamo et al., 2007), in the same way that Korean women with Pro/Ala + Ala/Ala alleles had a significant increase in body mass (*p* = 0.01), BMI (*p* = 0.01), and waist–hip ratio (*p* = 0.001) when compared to the Pro/Pro allele carriers in an intervention with diet and exercise (Kim et al., 2004).

In spite of this, there is no evidence to confirm the hypothesis that this gene acts positively or negatively in the lipolysis or slimming process, since it regulates the expression of numerous genes involved in lipid metabolism. Thus, the objective of this study was to verify the influence of the Pro12Ala polymorphism of the peroxisome proliferator-activated receptor gamma 2 (PPARγ2) on the weight loss induced by a continuous aerobic training program.

## Materials and Methods

This was a controlled and randomized clinical trial with 69 participants, of which 53 were genotyped and involved in an aerobic training program and 16 were part of the control group. In the case, the control group was formed to verify the effectiveness of the physical exercise, so they were not genotyped.

The sample size of this group was determined based on the study by Kim and Jung (2014), in which a similar training program resulted in a reduction of body fat by −2.18 ± 1.96%, and which implied an effect size of 1.11, which in turn generated a minimum sample size of 13 participants for a statistical β power of 0.95. To verify the effectiveness of the training program before starting comparisons between genotypes, a control group was set up that did not perform the training program with 16 people. The distribution of volunteers between the exercise and non-exercise groups was randomized.

Participants were invited through social media ads and pamphlets distributed near the study site, as well as medical clinics and community organizations. The detailed procedures are registered in the Clinical trials under identification NCT03568773.

In order to be eligible, participants had to be adults (ages 20–45 years), under active (<150 min/week of moderate to severe physical activity) as determined by the International Physical Activity Questionnaire (Matsudo et al., 2001), have a BMI of between 25 and 39.9 kg/m^2^ for at least 6 months, have not changed more than 5 kg in the last 3 months, did not smoke or consume alcohol (more than two doses/day), did not use medicine, supplements or thermogenic substances which alter the metabolism, and did not have any diseases (diabetes, coronary artery disease or hormonal diseases); for women, to not be menopausal or present any symptoms related to the climacteric period. Those who missed two consecutive weeks or 25% of the physical training program or who began dietary intervention, physical exercise, or medication during the program period, as well as those who were injured were excluded from the study.

All volunteers who agreed to participate in the study provided written consent after being clarified about procedures and potential risks. The experimental protocol was approved by the Human Research Ethics Committee of the Health Science Center-CCS of the Federal University of Paraíba-UFPB-Brazil, under protocol number 1.981.304.

### Study Design

The exercise group completed a 12-week aerobic training program and the control group did a stretching program over the same period. The volunteers underwent ergospirometry (aerobic capacity – VO_2_max and anaerobic threshold), Dual-energy X-ray absorptiometry (DXA) (body composition) dual-emission densitometry, abdomen, waist and hip circumference measures, glycemic and lipid profile (blood glucose, triglycerides, total cholesterol, HDL and LDL) and nutritional assessment (also in the sixth week of intervention) before and 48 h after the intervention periods. Each participant also had their buccal mucosa collected during the study for later genotyping.

### Aerobic Capacity and Anaerobic Threshold

Aerobic capacity and anaerobic threshold were performed by ergospirometry (Metalyzer 3B - Cortex (Leipzig-Germany) on a treadmill (Centurion-200 Micromed, Brasília – Brazil), with increasing load on a Bruce ramp protocol (8–12 min). Anaerobic Threshold (L1) and respiratory compensation point (L2) were recorded at the test time, which was registered immediately and then calculated for the mean time of 10 s determined at the exponentiation point of CO_2_, while VO_2_max was determined when the volunteers reached volitional fatigue accompanied by estimated HRmax. The criteria for interrupting the test followed the guidelines of Guazzi et al. (2018).

### Body Composition

Body composition was determined by Dual-energy X-ray absorptiometry (DXA) by a full body scan (brand: LUNAR ADVANCE DF + 13.4038 Radiation – GE LUNAR CORPORATION/United States), with the three compartment model (muscle, vital organs and other viscera of the body), fat tissue (amount of body fat) and bone tissue (total skeletal mass), being considered following the guidelines and calibration procedures provided by the manufacturer. In addition, weight, height (for evaluation of BMI) (a scale with coupled stadiometer, Sanny^®^, São Bernardo do Campo - São Paulo, Brazil) and the abdomen (greater abdomen circumference zone), waist (between the costal border and the iliac crest) and hip (maximum posterior hip extension) circumference measures were taken for analysis (Sousa, 2008).

### Blood Collection and Biochemical Measurements

Blood samples were collected in the antecubital vein early in the morning with the volunteer fasting for 10 h. They were then deposited in light-protected test tubes containing EDTA, homogenized by inversion and centrifuged at 3,000 rpm for 10 min. The supernatant was stored at −20°C until analysis. Lipid and Glycemic Profile analyzes were performed on serum samples using commercial kits from the Labtest brand (Minas Gerais, Brazil), following the manufacturer’s recommendations and on a Labmax 240 premium automatic analyzer (Lagoa Santa-MG, Brazil).

### Nutritional Control

Evaluations were performed before the intervention, in the sixth week and during the last week of intervention through the 24-h recall following a protocol suggested by Dietary Recommendation Intake (DRI) (Gibson, 1990). Three reminders were performed for each of the three evaluations, two of which were for weekdays and one for the weekend. AVANUTRI software version 4.0 (Avanutri & Nutrição Serviços de Informática, Três Rios-RJ-Brazil) was used for caloric and macro and micronutrient calculations. Volunteers were asked to not change their eating habits during the study after the pre-intervention evaluation.

### DNA Extraction and Genotyping

Oral epithelial cell samples were obtained with a 3% sucrose wash. DNA extraction was performed according to a previously published method (Aidar and Line, 2007). Genotyping of the Pro12Ala polymorphism (PPARγ2) was performed by the PCR-RFLP technique. The polymorphism was amplified using the primers: (5′-GCCAATTCAAGCCCAGTC-3′-sense and 5′-GATATGTTGGAGAGAGGGTATCAGTGAAGGAATCGCTTT CCG-3′-antisense). Thermal cycling was used as follows: initial denaturation at 94°C for 8 min and 35 denaturation cycles at 94°C for 50 s, annealing at 59°C for 50 s. and an extension at 72°C for 1 min, then final extension at 72°C for 5 min. After digestion with the BstU-I restriction enzyme (Biolabs, New England/United States), a single 270 bp fragment indicated the presence of the Pro/Pro genotype, while three 270, 227, and 43 bp fragments confirmed the presence of the Pro/Ala genotype. Lastly, 15% polyacrylamide gel electrophoresis and stained with silver nitrate was used for this genotypic reading.

### Physical Training Protocol

An adaptation protocol consisting of a 3-week treadmill (2 days/week, 20–40 min, intensity < L1 acquired in the ergospirometric test) was performed first. Next, the 12-week training program consisting of fast walking and/or running with intensity between the L1 and the L2 in an open-air environment was implemented as detailed in Table 1. There were 3 sessions/week from the first to the fourth week, 40–60 min, intensity = L1, always supervised by the researchers; in the fifth week the intensity increased between L1 and 1/2L2. There was an increase to five weekly sessions from the sixth to the eighth weeks, three of which were supervised by researchers and two in which the volunteers used an application (Endomondo Sports Tracker, version 17.5.1), and sent the report to the researchers. The duration was 60 minutes at this stage, with intensity between L1 and 1/2L2; then the time, volume and weekly frequency remained unchanged from the ninth to the twelfth weeks, and intensity increased to 1/2L2 to L2. Heart rate was continuously monitored in the laboratory sessions by heart rate monitors (Polar^®^, model FT1, Kempele, Finland). The subjects who were randomized to not participate in the training program (control group) participated in the same intervention period with stretching classes (1 day/week, duration of 60 min).

**TABLE 1:** Aerobic training protocol.

Week	Adaptation	1st	2nd and 3rd	4th	5th	6th to 8th	9th to 12th
Sessions/week	2	3	3	3	3	5	5
Time (min)	20 to 40	40	50	60	60	60	60
Intensity	<L1	L1	L1	L1	L1 a 1/2L2	L1 a 1/2L2	1/2L2 a L2

*Subtitle; <L1: below anaerobic threshold; L1 1/2 L2: between anaerobic threshold and half the respiratory compensation point; 1/2 L2 and L2: half of respiratory compensation point and respiratory compensation point.*

### Statistical Analysis

Data were expressed as mean ± standard deviation, or absolute values. The normality of data homogeneity was initially verified through the Kolmogorov–Smirnov and Levene tests, respectively. Two-way repeated measures ANOVA or Friedman test were used to compare the outcome of the training program between Pro/Pro and Pro/Ala genotypes. The Chi-squared test was performed to verify the genotype influence (Pro/Pro and Pro/Ala) on the variation in the body composition components, categorized as responders (Δ Weight: ≥1 kg; Δ BMI: ≥1 kg/m^2^; ΔFat mass: ≥1 kg; ΔLean mass: >0 kg; Δ Fat percentage: ≥1; Δ waist circumference: ≥2 cm; Δ hip circumference: ≥2 cm; Δ abdominal circumference: ≥2 cm) and non-responders (Δ Weight: <1 kg; Δ BMI: <1 kg/m^2^; ΔFat mass: <1 kg; Δlean mass: <0 kg; Δ Fat percentage: <1; Δ waist circumference: <2 cm; Δ hip circumference: <2 cm; Δ abdominal circumference: <2 cm) of the experimental group. A linear regression test was used starting with automatic linear modeling which considered the possible influencing variables (age, educational level, average daily sleep time, glycemic and lipid profiles, and nutritional behavior). Data analyzes were performed using the SPSS 20.0 Package (SPSS Inc., Chicago, United States) and a *p*-value of <0.05 was considered significant.

## Results

Of the 53 subjects in the trained group, 75.5% (*n* = 40) were identified as Pro/Pro and 24.5% (*n* = 13) with Pro/Ala genotype, with no Ala/Ala appearing in the sample. When the Hardy Weinberg Equilibrium was calculated considering *p* > 0.05, we observed that the study sample is consistent with the expected distribution (*p* = 0.31). When the Pro/Pro and Pro/Ala groups were compared, they were found to have similar ages, a physical activity level compatible with the insufficiently active classification (IPAQ) (Matsudo et al., 2001), and aerobic capacity between regular and weak according to the American Heart Association (American Heart Association, 1972), with no differences between groups. Likewise, they had similarity for all evaluated body composition components (Table 2).

**TABLE 2:** Baseline, variation anthropometric and biochemical characteristics of the participants in the experimental and control groups according to the Pro12Ala Gene PPARγ2 polymorphism.

Variables	Pro/Pro *n* = 40 (*n* = 13 males/*n* = 27 females)				Pro/Ala *n* = 13 (*n* = 2 males/*n* = 11 females)			Total Exercise *n* = 53 (*n* = 15 males/*n* = 38 females)			Non-exercise *n* = 16 (*n* = 3 males/*n* = 13 females)	
				
	Before	After	Δ	Before	After	Δ	Before	After	Δ	Before	After	Δ
N (%)	40 (75,5)			13 (24,5)			53 (76.8)			16 (23.2)		
Age (years)	33.27.5			33.09.2			33.17.6			29.18.8		
PA (min/Week)	75.329.4			71.532.4			72.69.2			69.128.4		
Sleep (hours/day)	7.01.4			6.01.4			6.61.5			6.12.1		
Glycemia (mg/dL)	96.818.8	95.220.5	−1.613.8	94.916.6	92.222.0	−2.710.3	96.318.2	94.520.7	−1.912.9	90.416.0	95.619.6	5.215.0
Col. Tot. (mg/dL)	199.848.8	183.337.1*	−16.533.0	183.741.1	164.155.6	−19.637.0	195.847.1	178.642.6*	−17.233.7	185.835.8	191.743.3	5.940.8^#^
HDL (mg/dL)	35.98.9	36.57.9	0.55.9	41.410.5	37.710.7	−3.67.5	37.39.5	36.88.6	−0.56.5	34.611.9	35.67.6	1.18.7
LDL (mg/dL)	131.640.9	120.833.9*	−10.731.9	120.334.2	104.755.5	−15.638.6	128.939.4	116.940.3*	−11.933.3	123.329.4	125.436.4	2.131.1
TG (mg/dL)	158.989.3	129.971.1*	−29.046.9	110.147.2	108.054.4	−2.179.6	146.983.4	124.567.6*	−22.457.0	139.975.7	153.684.8	13.761.5^#^
VO_2_max (mL⋅kg^−1^⋅min^−1^)	29.26.7	35.29.4	5.95.8	27.94.6	34.810.2	7.07.0	28.86.0	35.09.4	6.16.0	23.94.6^#^	24.82.7	0.22.6^#^
Weight (kg)	85.811.4	84.911.9	−0.82.7	82.110.3	80.99.4	−1.12.2	85.011.3	84.311.5	−0.82.5	82.711.2	83.212.1	0.42.1
BMI (kg/m^2^)	31.52.6	31.22.9	−0.31.2	31.23.2	30.82.9	−0.50.8	31.52.9	31.23.0	−0.31.1	31.02.9	31.13.2	0.10.8
FM (kg)	37.15.9	35.86.9*	−1.32.1	36.26.0	35.16.5*	−1.11.7	36.86.0	35.56.8	−1.31.9	38.47.6	38.28.4	−0.31.4^#^
LM (kg)	46.011.0	46.610.7*	0.61.5	41.67.0	41.96.7	0.31.2	45.210.1	45.89.9	0.61.5	41.56.8	44.511.4	2.99.5
F%	44.07.3	42.87.5*	−1.31.6	45.75.6	44.86.2*	−0.91.5	44.16.8	43.07.2	−1.21.5	46.75.8	46.15.9	−0.61.3
WC (cm)	94.17.5	91.97.2*	−2.22.9	90.68.2	89.69.2	−1.03.0	93.37.9	91.48.1	−2.02.8	93.88.7	93.99.8	0.23.6^#^
Abdomen (cm)	105.66.6	102.37.4*	−3.33.6	105.08.1	101.18.3*	−3.93.5	105.37.4	101.88.0	−3.53.6	103.810.4	104.210.7	0.54.7^#^
HC (cm)	110.50.6	107.76.2*	−2.72.7	112.07.0	110.26.1*	−1.81.8	110.76.0	108.36.3	−2.52.5	111.05.1	110.17.0	−0.53.1^#^

Data are means ± SD. N, number of participants; PA, Physical Activity; Col. Tot., total cholesterol; HDL, high density lipoprotein; LDL: low density lipoprotein; TG, triglycerides; BMI, Body Index Mass; FT, Fat Mass; LM, Lean Mass; F%, Fat Percentage; WC, Waist Circumference; HC, Hip Circumference. *Intra-group differences vs. initial values (pairwise Student’s *t-*test). #Between-groups differences (Two-Way ANOVA).

Although inclusion criteria predicted that diabetics could not participate in the study, people with borderline glycemic values were present in the sample, but there were no differences between the exercise and non-exercise groups (Table 2).

The Pro/Pro group had higher initial caloric intake, as well as macronutrients (carbohydrates and proteins) in comparison with Pro/Ala, as can be seen in Table 3. The evaluations performed in the sixth week and at the end of the protocol indicated that the two groups maintained the same feeding profile in relation to the initial values.

**TABLE 3:** Intake of macronutrients and micronutrients of participants within 12 weeks of intervention.

Nutrition	Pro/Pro			Pro/Ala			*p*
		
	Baseline	6th week	12th week	Baseline	6th week	12th week	
Energy (kcal)	1947.5580.0^#^	1924.0598,2	1885.1564.1	1535.3412.3	1562.8360.6	1720.6466.6	0.02
Carbohydrate (g)	253.377.8^#^	247.984.0	235.874.4	203.767.7	193.747.5	223.699.6	0.04
Total fat (g)	63.020.6	63.725.1	66.723.1	51.317.0	52.520.8	59.218.9	
Protein (g)	89.241.3^#^	89.835.4	88.332.8	64.715.4	78.936.8	73.527.9	0.02
SFA (g)	17.37.0	18.48.7	18.16.8	15.010.9	15.06.1	17.68.0	
MUFA (g)	14.86.0	14.96.5	15.66.9	11.24.5	13.87.9	14.85.3	
PUFA. (g)	9.75.4	10.26.1	9.24.5	7.23.7	8.65.6	10.24.8	
Cholesterol (mg)	309.3212.1	338.6229.5	312.1172.6	223.452.0	286.4125.5	295.4123.2	
Fibers (g)	14.67.0	16.47.7	15.18.4	11.64.2	11.53.6	11.84.7	

*Data are means ± SD. SFA, Saturated Fatty Acids; MUFA, Monounsaturated Fatty Acids; PUFA, Polyunsaturated fatty acids. ^#^Differences between groups Pro/Pro vs. Pro/Ala, initial values. *P* < 0.05. (Two-way ANOVA and Friedman’s test).*

The previous analysis comparing study participants with a sample of people who did not participate in the training program confirmed that exercise promoted increased aerobic capacity in the trained group, which was accompanied by a significant reduction in all body composition components related to obesity and increased muscle mass, in addition to reducing total cholesterol, its LDL fraction and triglycerides, without the same occurring in the group that did not exercise. Glycemia and HDL cholesterol fraction did not change in either group (Table 2).

### Weight Loss According to Genotype (Pro/Pro versus Pro/Ala)

**FIGURE 1:**
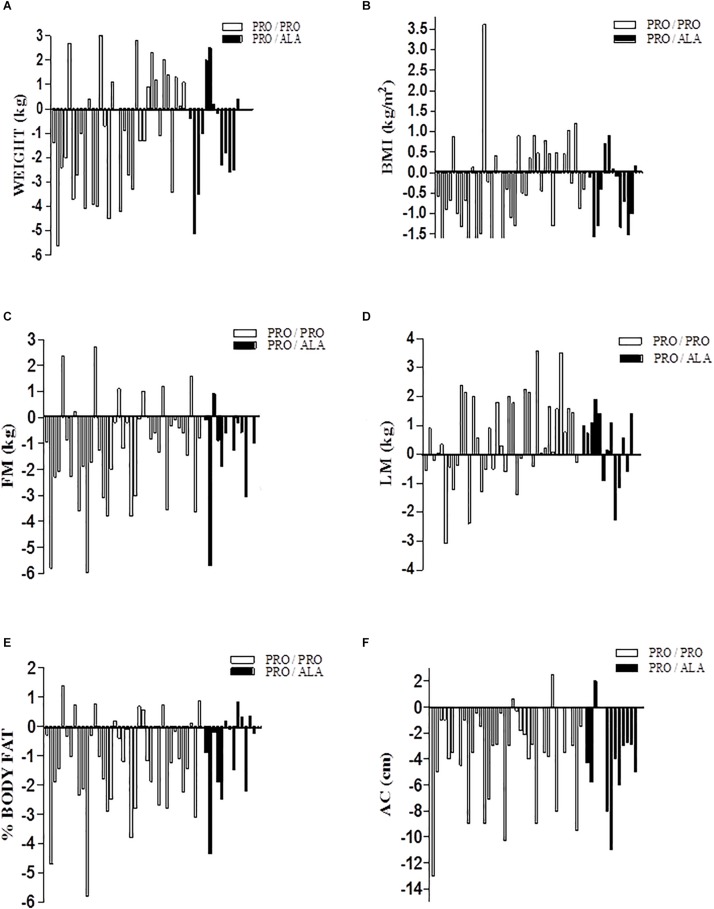
Individual response of body composition to aerobic training between genotypes. Date are: variation of final value minus initial value (Δ variation); **(A)** body weight; **(B)** Body mass index; **(C)** fat mass; **(D)** lean mass; **(E)** body fat percentage; **(F)** abdominal circumference.

Figure 1 shows a consistent individual variability in the weight loss response and in the increase of lean mass in the training program. As can be seen in panel C which corresponds to fat mass, there were people who reduced 6.0 kg, but others increased up to 2.7 kg of fat at the end of the training program. This same variability occurred for the other body composition components and are presented in Figure 1.

The data shown in Table 2 indicate a slight weight-loss superiority in volunteers with Pro/Pro genotype, since this group had a significant reduction in fat mass, fat percentage, and abdomen and hip circumferences, in addition to the increase in lean mass. Meanwhile, Pro/Ala genotypes showed no significant reduction in waist circumference and no increase in lean mass. However, the chi-squared test indicated that the studied genotype was not a determinant of greater or lesser weight loss for any of the body composition variables (Table 4).

**TABLE 4:** Association test between PPARγ2 gene polymorphism and responders and non-responders to weight loss training program.

Dependent variables	PPARγ2		Total	*p*
		
	Pro/Pro *n* (%)	Pro/Ala *n* (%)	*n*	
Δ Weight				
Responders	23 (57.5)	9 (69.2)	32	0.447
Non-responders	17 (42.5)	4 (30.8)	21	

Total	40	13	53	

ΔBMI				
Responders	23 (57.5)	8 (61.5)	31	0.797
Non-responders	17 (42.5)	5 (38.5)	22	
Total	40	13	53	

ΔFM				
Responders	26 (65.0)	10 (76.9)	36	0.414
Non-responders	14 (35.0)	3 (23.1)	17	
Total	40	13	53	

ΔF%				
Responders	31 (77.5)	8 (61.5)	39	0.257
Non-responders	9 (22.5)	5 (38.5)	14	
Total	40	13	53	

ΔLM				
Responders	25 (62.5)	9 (69.2)	34	0.658
Non-responders	15 (37.5)	4 (30.8)	19	
Total	40	13	53	

ΔWC				
Responders	29 (72.5)	9 (69.2)	38	0.821
Non-responders	11 (27.5)	4 (30.8)	15	
Total	40	13	53	

ΔAC				
Responders	33 (82.5)	10 (76.9)	43	0.661
Non-responders	7 (17.5)	3 (23.1)	10	
Total	40	13	53	

ΔHC				
Responders	28 (70.0)	7 (53.8)	28	0.228
Non-responders	12 (30.0)	6 (46.2)	25	
Total	40	13	53	

*Date are: frequency of responders (who obtained some weight loss) and non-responders (who did not lose weight or increased any variable related to weight loss. BMI, Body Index Mass; FT, fat mass; LM, lean mass; F%, fat percentage; WC, waist circumference; HC, hip circumference; AC, abdominal circumference. The Chi-squared test (McNemar test) was used to observe the frequency and Fisher’s test for values below five.*

Considering that the Chi-squared test ruled out the genotypic influence on exercise-induced weight loss, we performed a linear regression to verify that other variables could be influential. Other variables such as vitamin C (β = 0.41; *p* = 0.00), vitamin D (β = 0.33; *p* = 0.00) and potassium were found to be positively correlated, while gender (β = 0.28; *p* = 0.00), hours of sleep (β = −0.37; *p* = 0.00), Magnesium (β = −0.81; *p* = 0.00), Zinc (β = −0.34; *p* = 0.00), Iodum (β = −0.04; *p* = 0.03) and Fibers (β = −0.31; *p* = 0.03) were negatively correlated. It seems that, when adjusted by gender, these influences have disappeared. Therefore, although some influence was noticed, they were consistency weak for the variables of body composition that were analyzed, especially when adjusted for gender (Supplementary Table S1).

## Discussion

Discrete weight loss in response to physical training programs has been highlighted in the literature since the middle of the first decade of this century (Douketis et al., 2005; Johns et al., 2014). Although the participants were significantly thinner with the training program, weight loss observed in this study can also be considered clinically discreet (−0.9 ± 2.6 kg, −1.3 ± 2.0 kg, and −1.2 ± 1.6% for body weight, body fat and fat percentage respectively). These data are similar to reviews and meta-analyzes (Johns et al., 2014; Washburn et al., 2014) with physical exercise-induced weight loss which did not analyze the genetic influence. They are also similar to those found in other studies that investigated the influence of PPARγ2 on weight loss (Østergård et al., 2005; Franks et al., 2007; Delahanty et al., 2012).

In addition to corroborating literature on the magnitude of physical exercise, the data from our study corroborate previous literature, indicating significant individual variability in weight loss (Donnelly et al., 2013). The authors of this study showed that only 62.2% of participants, including men and women, achieved weight loss. This individual variability indicates that personal characteristics (which may be physiological, genetic or otherwise) are influential in the weight-loss response to physical training. The influence of the PPARγ2 gene on both obesogenesis and weight loss is still controversial. Regarding obesogenesis, some studies have shown an association of the risk allele with BMI or other variables related to obesity (Danawati et al., 2005; Franks et al., 2007; Gupta et al., 2011; Bhatt et al., 2012), but this association was not found in other population studies (Ereqat et al., 2009; Wang et al., 2014). The data for the influence on weight loss are incipient, while some studies with diet interventions show that Ala allele carriers have dietary resistance (Nicklas et al., 2001; Adamo et al., 2007). On the other hand, Verhoef et al. (2014) demonstrated that Pro/Ala was positively associated with short-term weight loss.

Data related to weight loss in response to training programs until the present study were incipient, but corroborating. In the present study, it was found that the Ala allele had a high affinity for alleles; therefore, our data are controversial regarding the relationship of the PPARγ2 gene with exercise-induced weight loss, as it already occurs in the areas of obesogenesis (Franks et al., 2007; Gupta et al., 2011; Bhatt et al., 2012) and diet-induced weight loss or lifestyle modification (Nicklas et al., 2001; Adamo et al., 2007).

Methodological differences in the interventions of the previous studies are likely to explain the differences in results in our study compared to the three previous studies. In two of them, the intervention consisted of physical exercise in the form of lifestyle modification, so that people started to perform 150 min of physical activity/week (Franks et al., 2007; Delahanty et al., 2012). This differs greatly from our study, in which the intervention was a systematic physical training program. The only study in which a similar protocol was performed was that of Østergård et al. (2005), where participants underwent aerobic training (three times a week, 45 minutes of duration at 70% VO_2_ max) for 10 weeks; but the population was constituted by relatives of first-degree diabetics and using a stationary bicycle.

Although our study found no influence of the Pro12Ala polymorphism of the PPARγ2 gene on weight-induced exercise, we presented some practical implications, for research laboratories. We observed that sleep hours and nutritional aspects were shown to be influencers in the promoted weight loss, although without much consistency for the analyzed body composition variables. In any case, the nutritional differences between Pro/Pro and Pro/Ala in our findings could have occurred because the homozygous group had a larger number of men in the sample, although this was not an influencing factor in linear modeling when adjusted for gender.

This reinforces the multifactorial and complex aspect of weight loss, so that other variables which were not considered in this study such as the metabolic profile, lipolytic or adipogenic hormones, fiber type, and behavioral aspects which have been hypothesized as influencers of weight loss (Boutcher and Dunn, 2009) should be considered in future studies, in which genetics may contribute to elucidate the relationship of discrete exercise-induced weight loss.

In addition to the physiological multiplicity, this same perspective must be kept in mind for the multiplicity of genes (besides PAPR) which may be involved in weight loss. In addition, it is necessary to investigate other genes (isolated or associated) which have been demonstrated to be involved in obesity such as FTO (Ben Halima et al., 2018), Melanocortin 4 Receptor - MC4R (Resende et al., 2017), Adenovirus 36 (Zhou et al., 2018) and beta 2 adrenoceptor-ADRB2 (Daghestani et al., 2018).

## Conclusion

It was demonstrated that Pro12Ala polymorphism in the PPARγ2 gene does not influence the magnitude of the weight loss induced by aerobic training. Other genes, other physiological factors, as well as a larger number of volunteers participating in a training program should be considered in future studies. There must also be at least one group in which Ala/Ala alleles appear within the investigated sample.

## Data Availability Statement

All datasets generated for this study are included in the article/Supplementary Material.

## Ethics Statement

The studies involving human participants were reviewed and approved by Human Research Ethics Committee of the Health Science Center-CCS of the Federal University of Paraíba-UFPB-Brazil, under protocol number 1.981.304. The patients/participants provided their written informed consent to participate in this study.

## Author Contributions

GC and AS conceived the idea for the manuscript, agreed on content, contributed to the writing and editing the manuscript, and approved the final draft of the manuscript. DP, MR, BS, KF, AA, JM-F, and RS conceived the editing the manuscript, and approved the final draft of the manuscript.

## Conflict of Interest

The authors declare that the research was conducted in the absence of any commercial or financial relationships that could be construed as a potential conflict of interest.
