# Supplementary material for: Pro12Ala Polymorphism on the PPARγ2 Gene and Weight Loss After Aerobic Training: A Randomized Controlled Trial
Source: Front Physiol. 2020 May 8;11:385. doi: 10.3389/fphys.2020.00385 (PMC7227424; doi:10.3389/fphys.2020.00385)
Supplement: Supplementary file 1 [file Table_1.docx]

| **Table S1.** Variables involved in induced exercise weight loss with linear regression | | | | | | | | | | | | |
| --- | --- | --- | --- | --- | --- | --- | --- | --- | --- | --- | --- | --- |
|  | △ Weight | | △ BMI | | △ FM | | △ F% | | △ LM | | △ AC | |
|  | Beta | Sig. | Beta | Sig. | Beta | Sig. | Beta | Sig. | Beta | Sig. | Beta | Sig. |
| Age (years) |  | - | - | - | 0.36 | 0.00 | - | - | - | - | -0.29 | 0.03 |
| Gender | 0.28 | 0.00 | - | - | - | - | - | - | - | - | - | - |
| Sleep (hours / day) | -0.37 | 0.00^*^ | - | - | - | - | -0.38 | 0.00^*#^ | - | - | - | - |
| Triglycerides (mg / dL) Pré | - | - | - | - | 0.46 | 0.00 | 0.31 | 0.02^*#^ | - | - | - | - |
| RHR_ Pré | - | - | - | - | - | - | - | - | - | - | -0.55 | 0.00^*^ |
| VO_2_max (mL/kg/min)_Pré | - | - | - | - | - | - | 0.28 | 0.03^#^ | - | - | - | - |
| Hip (cm) _Pre | - | - | 0.31 | 0.02 | 0.25 | 0.04 | - | - | - | - | - | - |
| Vitamin C(mg)_Pré | 0.41 | 0.00^*#^ | - | - | - | - | - | - | 0.36 | 0.02^*^ | - | - |
| Vitamin C (mg)_ 6^th^ | -0.28 | 0.03^#^ | - | - | - | - | - | - | -0.25 | 0.09 | - | - |
| Vitamin D (mg)_Pré | - | - | - | - | - | - | - | - | - | - | 0.26 | 0.03^*^ |
| Vitamin D (mg)_ 6^th^ | 0.33 | 0.00 | - | - | - | - | - | - | - | - | - | - |
| Vitamin E (mg)_Pré | - | - | - | - | 0.29 | 0.02 | - | - | - | - | - | - |
| Ca_ Pré | - | - | - | - | - | - | - | - | -0.43 | 0.00 | - | - |
| Na_ 6^th^ | - | - | - | - | -0.58 | 0.00^*^ | - | - | - | - | - | - |
| Mg_ Pré | -0.81 | 0.00^*#^ | - | - | - | - | - | - | - | - | - | - |
| Zn_ Pré | -0.34 | 0.00^*^ | - | - | - | - | - | 0.35 | - | - | -0.24 | 0.04^*#^ |
| K_ Pré | 0.95 | 0.00^#^ | - | - | - | - | - | - | - | - | - | - |
| I_ Pré | -0.24 | 0.03 | -0.37 | 0.01 | - | - | - | - | - | - | - | - |
| Fibers (g)_ Pré | -0.31 | 0.03^#^ | - | - | - | - | - | - | - | - | - | - |
| Selenium - 6^th^ | - | - | - | - | - | - | -0.38 | 0.02^*#^ | - | - | - | - |
| Saturated Fat (g) _ Pre | - | - | - | - | -0.22 | 0.07 | - | - | - | - | -0.33 | 0.01 |
| Polyunsaturated fats (g) _6^th^ | - | - | - | - | 0.30 | 0.04 | - | - | - | - | - | - |
| Date are: BMI: Body Index Mass; FT: Fat mass; LM: Lean Mass; F%: Fat Percentage; AC; abdominal circumference. LDL: low density lipoprotein; RHR: resting heart rate; Ca: calcium; Na: sodium; Mg: magnesium; Zn: zinc; K: potassium; I: iodine.  p <0.05: entered the model and proved to be intervening  p> 0.05 entered the model, but it was neither significant nor intervening  Independent variable with sign “-“ indicates that it has not entered the linear regression model for the dependent variable in question  * p <0.05 when adjusted for females  #p <0.05 when adjusted for males | | | | | | | | | | | | |
